# Evaluation of intake of aged garlic extract and organosulfur compounds on progressive hearing loss in DBA/2J mice

**DOI:** 10.1371/journal.pone.0322105

**Published:** 2025-04-23

**Authors:** Hideaki Oike, Kyoko Yamasaki, Kenji Hashiguchi, Honoka Uehira

**Affiliations:** 1 Institute of Livestock and Grassland Science, National Agriculture and Food Research Organization (NARO), Tsukuba, Ibaraki, Japan; 2 Research and Development Department, Momoya Co., Ltd., Tokyo, Japan; University of the Pacific, UNITED STATES OF AMERICA

## Abstract

Garlic is rich in organosulfur compounds with high antioxidant capacity and is known to have various health benefits. Aged garlic is a particularly effective source of these active compounds because it contains fewer toxic components. This study evaluated the effects of S-allyl cysteine (SAC), a major functional organosulfur compound in garlic, and aged garlic extract (AGE) on the suppression of progressive hearing loss. SAC and AGE were dissolved in drinking water at 1% (w/w) and administered to DBA/2J mice, a model of early progressive hearing loss, for 12 weeks, starting at 4 weeks of age. While the results revealed a trend toward weight loss in the SAC group, the weight of the AGE group was comparable to that of the control group, and no adverse events were observed in either group. The hearing ability of the mice was measured using auditory brainstem responses at 4, 8, 12, and 16 weeks of age. Hearing loss at 8 and 16 kHz progressed over the 12-week period, with neither sample inhibiting hearing loss. In contrast, another organosulfur compound, N-acetylcysteine (NAC), administered in 1% w/w drinking water and evaluated for hearing loss over time, significantly suppressed hearing loss progression in DBA/2J mice. These results indicate that the NAC and SAC differ in their ability to prevent hearing loss and demonstrate that the inhibitory effects of functional food components on hearing loss can be evaluated over a short period in DBA/2J mice.

## Introduction

According to WHO’s World Hearing Report, about 2.5 billion people worldwide (one in four) will have hearing loss by 2050. The report also noted that at least 700 million people require preventive measures. Even among younger generations, it is estimated that more than 1.4 billion people are at risk of hearing loss because of unsafe sound-listening habits [1].

There is considerable individual variation in the progression and susceptibility to hearing loss, with over 100 identified genes involved in hearing loss [2]. Hearing evaluations in mice have long been used in genetic research to study hearing loss [3–5]. Therefore, different mouse strains exhibit different auditory characteristics. The DBA/2J mouse strain shows early progressive hearing loss and has been used as an animal model of hearing loss [6].

N-acetylcysteine (NAC), a sulfur-containing amino acid with antioxidant properties, is expected to reduce noise-induced hearing loss [7,8]. S-Allylcysteine (SAC) is another sulfur-containing amino acid with antioxidant properties that exhibits diverse biological and pharmacological effects, including cancer prevention, neuroprotection, hepatoprotection, antidiabetic, cardioprotective, anti-asthma, and renoprotection [9–11]. SAC crosses the blood-brain barrier in animal experiments using rodents and is expected to improve brain function [12]. Indeed, aged garlic extract (AGE) rich in SAC has antioxidant properties [11,13,14] and has been shown to improve sleep quality and reduce fatigue in human studies [15,16], as well as improve peripheral blood flow and restore peripheral body temperature due to cold [17]. In the present study, we investigated the preventive effects of SAC, AGE, and NAC on hearing loss in DBA/2J mice.

## Materials and methods

Animals were handled in accordance with the guidelines of the Japanese Ministry of Agriculture, Forestry, and Fisheries for laboratory animal studies. This study was reviewed and approved by the Animal Care and Use Committee of the Food Research Institute, NARO (approval numbers: 20B180FRI and R4-M17-NILGS).

**Fig 1:**
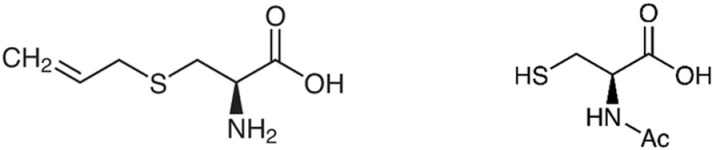
Chemical structures of SAC (left) and NAC (right).

DBA/2J mice (male, 3 weeks old) were obtained from CLEA Japan, Inc. (Tokyo, Japan) and housed at 25 ± 1 °C with a 50 ± 5% humidity and a 12 h light–dark photocycle with ad libitum access to food and water, four mice per cage. AGE was produced by heat-aging a water extraction of garlic using the method described in Japanese Patent No. 5968729 (Momoya; SAC 237 mg/100 g, analyzed according to a previous report [18]). AGE, SAC (Tokyo Chemical Industry, Tokyo, Japan), and NAC (Sigma-Aldrich, St. Louis, MO, USA) were administered dissolved in tap water at 1% (w/w). AGE was filter-sterilized before being dissolved in drinking water and the solution was replaced within two days to avoid bacterial growth. The chemical structures of SAC and NAC are shown in Fig 1.

The hearing ability of mice was assessed using an auditory brainstem response test, as previously described [19]. Briefly, the mice were intraperitoneally anesthetized with a mixture of medetomidine (0.3 mg/kg), midazolam (4.0 mg/kg), and butorphanol (5.0 mg/kg), and subdermal needle electrodes were placed at the vertex (reference), beneath the pinna of both ears (active), and lower back (ground). The mice were placed on a warming mat during anesthesia, and measurements were taken to prevent a drop in body temperature. The sound stimulus consisted of a 5 ms tone burst with a rise-fall time of 1.5 ms at frequencies of 8 and 16 kHz (10–100 dB sound pressure limit (SPL)). The responses to 500 sweeps were averaged at each intensity level (5 dB SPL steps) to assess the threshold. Hearing threshold was defined as the lowest stimulus intensity that produced reliable peaks in the auditory brainstem response (ABR) waveforms. For convenience, if no peak was observed (even at 100 dB), a 105 dB SPL was recorded as the threshold. Better scores were recorded as the thresholds between the right and left ears. Mice were euthanized under deep anesthesia after blood sampling.

## Results

**Fig 2:**
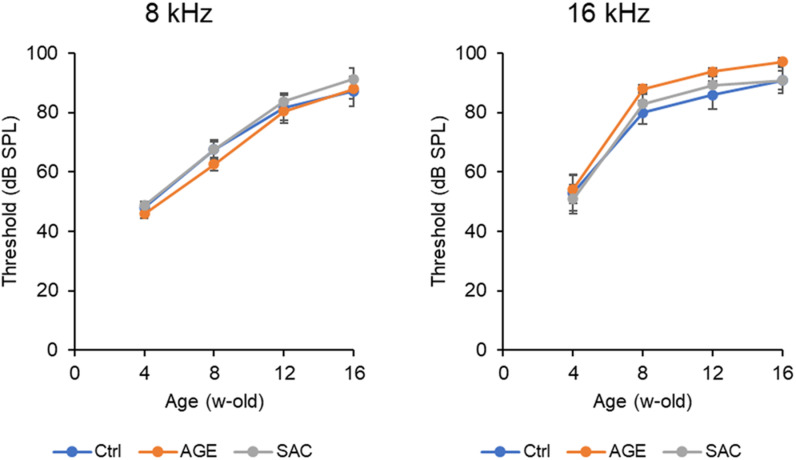
Age-related changes in hearing thresholds in DBA/2J mice (SAC and AGE study).

First, SAC and AGE were administered. The hearing thresholds for the 8 and 16 kHz pure-tone stimuli were approximately 50 dB at 4 weeks of age, prior to the administration of the test samples, but increased to approximately 60–70 dB at 8 weeks of age for 8 kHz and 80–90 dB at 16 kHz. Furthermore, at the age of 12 weeks, those values increased to approximately 80 and 90 dB at 8 and 16 kHz, respectively. At 16 weeks, both the 8 and 16 kHz reached approximately 90 dB (Fig 2). No significant differences (one-way ANOVA) were detected among the three groups (control, SAC, and AGE) at any age.

**Fig 3:**
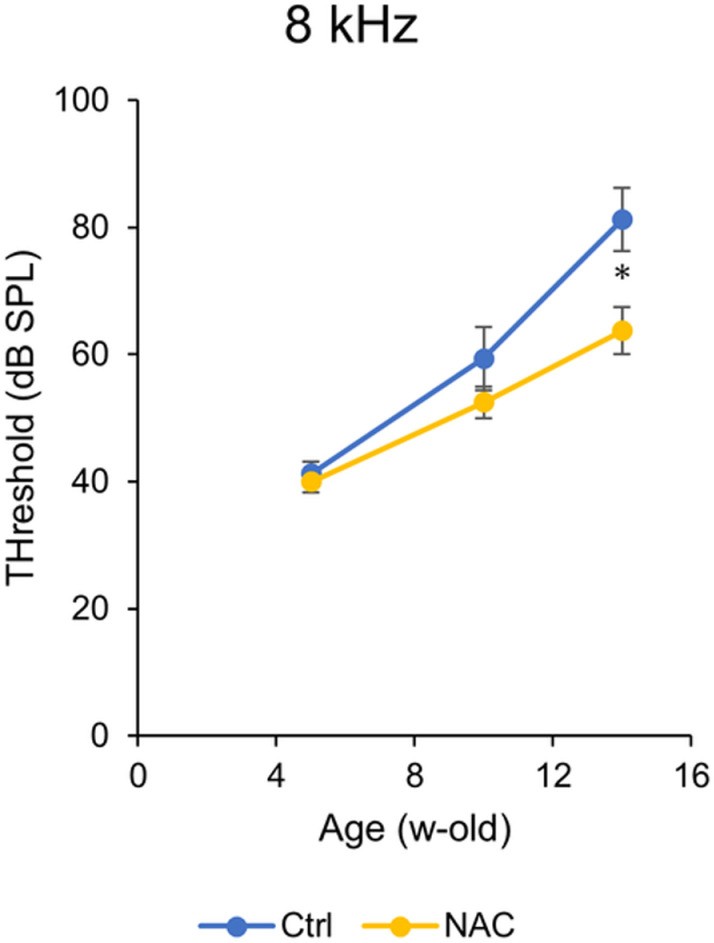
Age-related changes in hearing thresholds in DBA/2J mice (NAC study).

In the subsequent NAC administration study, the 8 kHz threshold was approximately 40 dB at 5 weeks of age before the test sample was administered, increasing to 50–60 dB at 10 weeks and 60–80 dB at 14 weeks of age (Fig 3), and significant differences between the control and NAC groups were detected at 14 weeks of age (p < 0.05, t-test).

In addition, the SAC and NAC groups tended to consume less water and had lower body weights (S1 and S2 Figs). The mean intake of SAC, AGE, and NAC throughout the study period was approximately 1.0, 1.3, and 0.9 g/kg body weight/day, respectively.

## Discussion

NAC suppressed hearing loss in DBA/2J mice, a model of early hearing loss progression, whereas SAC and AGE did not. This study provided several key insights. First, while both NAC and SAC are organosulfur compounds with high antioxidant capacities, they exhibit different hearing loss suppression effects. Second, although raw garlic is toxic to many animals and should not be consumed directly, AGE did not produce any adverse events or effects during the 12-week feeding trial, making it an excellent source of organosulfur health-function components. Third, the ability of NAC to suppress hearing loss in DBA/2J mice suggests that this model can be used to search for hearing loss-suppressing substances and mechanisms in a shorter period compared to other rodent models.

SAC, like NAC, is a cysteine derivative with strong antioxidant properties. It is an organosulfur compound found in garlic and has been reported to have various health benefits [11]. In particular, hearing loss due to ototoxic substances is prevented by garlic derivatives containing SAC [20]. The mechanisms underlying the health effects of these garlic derivatives include a reduction in reactive oxygen species levels due to their antioxidant capacity and an increase in stress tolerance via the induction of Nrf2 due to their hormesis effect [21,22]. It is water-soluble, stable in the blood, accessible to various tissues, and exhibits high oral bioavailability. However, in the present study, NAC prevented hearing loss, whereas SAC and AGE, with high SAC content, did not. This suggests a difference in the mechanisms of action between NAC and SAC. The concentrations of SAC tested in this study were comparable to those that were protective against liver damage in rodent studies [23,24] and were probably physiologically effective. Therefore, the inhibitory effects of NAC on progressive hearing loss could not be explained by its antioxidant capacity. It has been argued that the cytoprotective activity of NAC cannot be explained solely by its antioxidant capacity because the action of glutathione supplementation in cells is important [25,26]. In contrast, it has been suggested that the health effects of AGE involve increased blood glutathione peroxidase activity [15]. AGE containing SAC has also been reported to reduce muscle damage and improve chronic inflammatory responses by decreasing oxidative stress when consumed by humans subjected to excessive exercise [27]. SAC also crosses the blood-brain barrier [28], and in rats subjected to restraint stress, it has been shown to protect the brain from oxidative damage caused by acute stress [29]. Thus, NAC and SAC have potent antioxidant activities in vivo; however, their mechanisms of action are different.

In the present study, SAC, SAC-rich AGE, and NAC were administered in 1% drinking water. Although we did not obtain exact values for the amount of water consumed per animal because water consumption was measured in cage units, the amount of water consumed by SAC and NAC was approximately 30–50% lower than that of AGE and the control. This may be due to an aversive taste or odor. AGE contains high levels of organosulfur compounds but its pungent odor and irritant molecules are reduced during maturation, making it less toxic than raw garlic [11]. This could be interpreted as a reduction in the aversiveness of mice due to drinking water administration. Because AGE can be consumed with fewer unpleasant components than raw garlic, it is envisioned that it can be used in foods consumed daily to prevent lifestyle-related diseases.

Consistent with previous reports [3], the hearing threshold of DBA/2J mice increased with age in the high-frequency range (16 kHz), followed by an increase in the threshold in the low-frequency range (8 kHz). This indicates that progressive hearing loss occurred during the study period, as expected. The reproducibility of the assay system was confirmed to be acceptable as the two studies showed similar hearing loss progression time courses.

NAC suppresses age-, noise-, and drug-induced hearing loss in rodent models [30–35]. In contrast, another study reported that NAC did not suppress early age-related hearing loss in C57BL/6J mice [36]. Although the mechanism of action is thought to involve a direct or indirect antioxidant capacity, this is still under consideration. Confirmation of the efficacy of NAC in preventing progressive hearing loss in DBA/2J mice has several benefits. DBA/2J mice show a faster time course for hearing loss progression than other mouse strains or rodent models, allowing a shorter time to conduct studies to elucidate the mechanism of action. It is also a useful test system for identifying the components of hearing protection other than the NAC.

The vertical axis indicates the auditory threshold determined from auditory brainstem responses. The horizontal axis indicates week-old mice. Absolute sound pressure, mean ± standard error, 12 mice per group.

(Left) Responses to an 8 kHz pure tone increased linearly up to 12 weeks of age, with a slight plateau at 16 weeks of age. (Right) Responses to 16 kHz pure tones had already plateaued after 8 weeks of age, indicating that hearing loss progressed earlier than that at 8 kHz, and there were no significant differences between the three groups.

The vertical axis indicates the auditory threshold determined from auditory brainstem responses. The horizontal axis indicates week-old mice. Shown as absolute sound pressure, mean ± standard error, eight mice per group. Drinking water administration of NAC (1% w/w) significantly suppressed week-dependent progressive hearing loss (* p < 0.05; t-test).

## Supporting information

S1 Fig Body weight, food intake and water consumption in the SAC and AGE study. Each graph shows the changes in body weight (A), food intake (B), and water consumption (C) during the study period. Food intake and water consumption are shown as measured per cage and divided by the number of animals. Body weights are shown as mean ± standard error.
(TIF)

S2 Fig Body weight, food intake and water consumption in the NAC study. Each graph shows the changes in body weight (A), food intake (B) and water consumption (C) during the study period. Food intake and water consumption are shown as measured per cage and divided by the number of animals. Body weights are shown as mean ± standard error.
(TIF)

S1 Table Hearing thresholds of individual mice in the SAC and AGE study. (XLSX)

S2 Table Hearing thresholds of individual mice in the NAC study. (XLSX)
