# Supplementary figures and images for: Evaluation of intake of aged garlic extract and organosulfur compounds on progressive hearing loss in DBA/2J mice
Source: PLoS One. 2025 Apr 23;20(4):e0322105. doi: 10.1371/journal.pone.0322105 (PMC12017496; doi:10.1371/journal.pone.0322105)

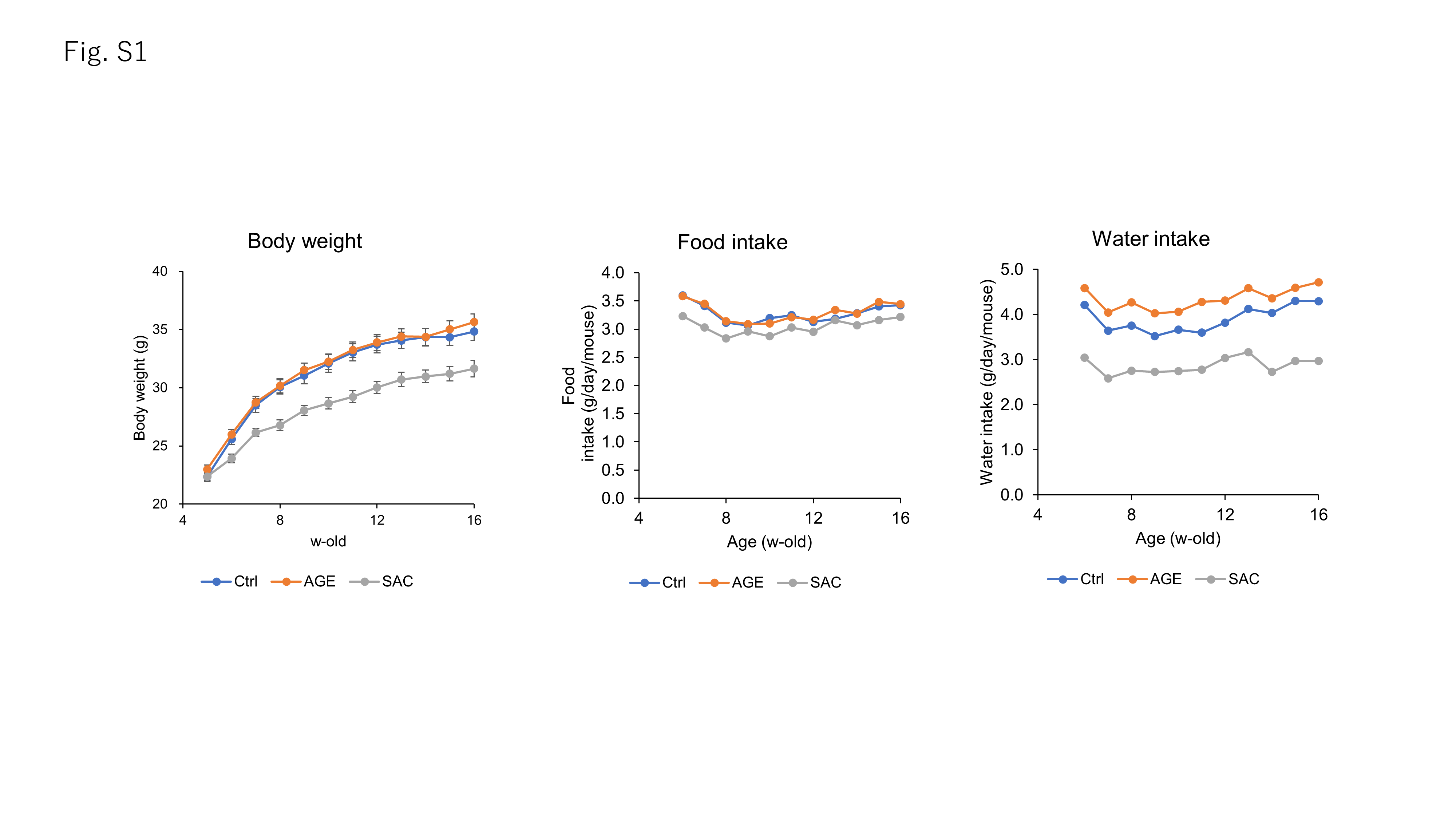

Supplement: S1 Fig — Each graph shows the changes in body weight (A), food intake (B), and water consumption (C) during the study period. Food intake and water consumption are shown as measured per cage and divided by the number of animals. Body weights are shown as mean ± standard error. (TIF) [file pone.0322105.s001.TIF]

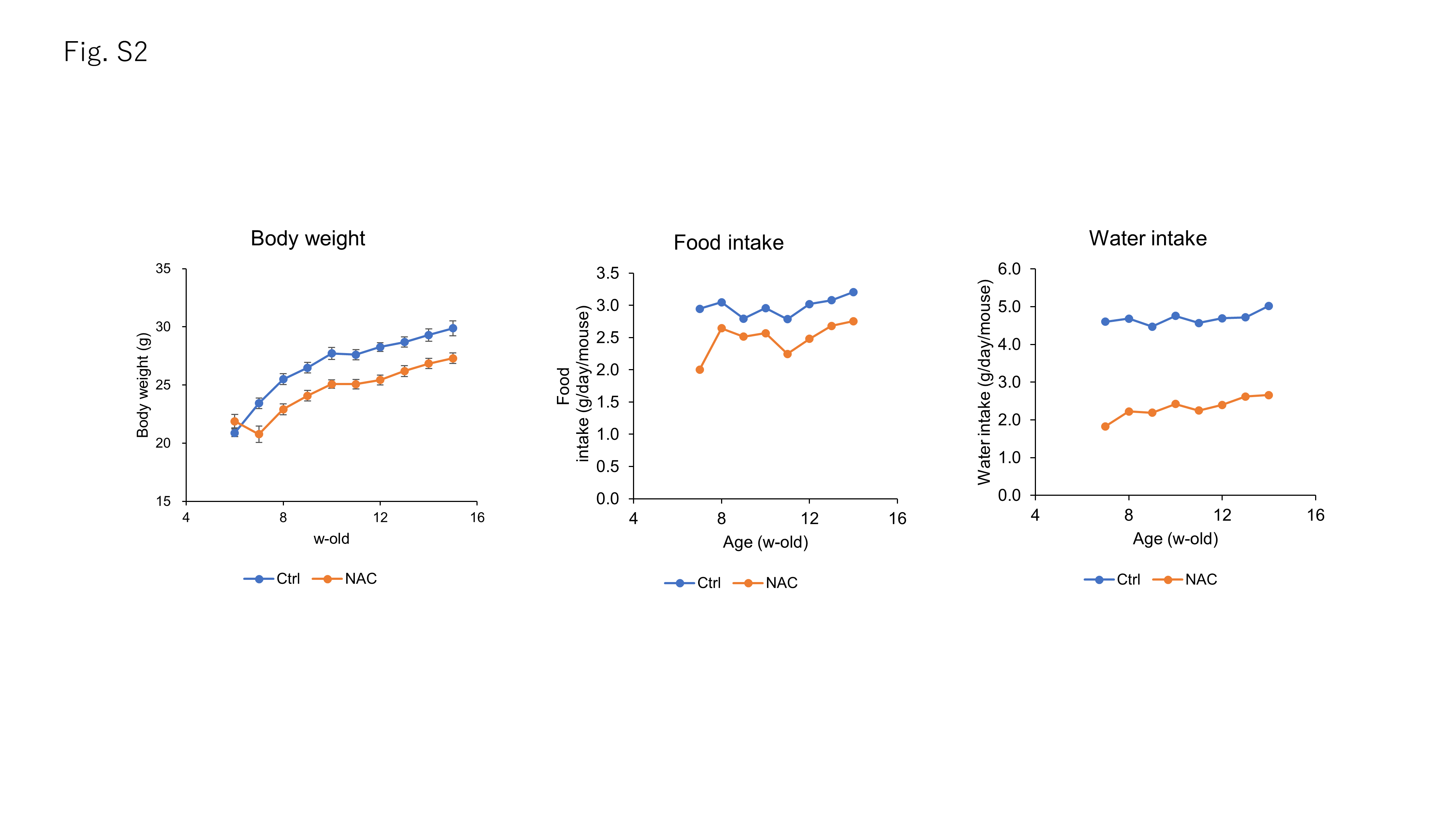

Supplement: S2 Fig — Each graph shows the changes in body weight (A), food intake (B) and water consumption (C) during the study period. Food intake and water consumption are shown as measured per cage and divided by the number of animals. Body weights are shown as mean ± standard error. (TIF) [file pone.0322105.s002.TIF]
